# A Japanese family with cone-rod dystrophy of delayed onset caused by a compound heterozygous combination of novel *CDHR1* frameshift and known missense variants

**DOI:** 10.1038/s41439-019-0048-8

**Published:** 2019-04-12

**Authors:** Muhammad Nazmul Haque, Kentaro Kurata, Katsuhiro Hosono, Masafumi Ohtsubo, Kentaro Ohishi, Miho Sato, Shinsei Minoshima, Yoshihiro Hotta

**Affiliations:** 1 Department of Photomedical Genomics, Institute for Medical Photonics Research, Preeminent Medical Photonics Education and Research Center, Hamamatsu University School of Medicine, Hamamatsu-shi, Shizuoka-ken Japan; 2 Department of Ophthalmology, Hamamatsu University School of Medicine, Hamamatsu-shi, Shizuoka-ken Japan

**Keywords:** PCR-based techniques, Genetics research

## Abstract

We analyzed two siblings in a Japanese family with delayed onset cone-rod dystrophy (CRD) using whole-exome sequencing. A novel frameshift c.1106dup (p.H370Afs*17) variant and a known missense c.2027 T > A (p.I676N) variant in *CDHR1* were identified. Both patients shared the same variants, although they displayed a significant difference in disease severity. A meta-analysis of the relationship between the severity and the variant type was performed using the reported cases in the literature and did not reveal a definitive correlation.

CRD is an inherited retinal disease in which the cones are predominantly damaged earlier than the rods. CRD eventually leads to severe visual impairment^1–3^. Cadherin-related family member 1 (*CDHR1*; NM_033100.3; NP_149091.1) is 1 of the 34 reported causal genes for CRD listed in RetNet^TM^^4^ and is described in OMIM as CONE-ROD DYSTROPHY 15 (MIM # 613660; https://omim.org/). CDHR1 protein is expressed in several tissues, most abundantly in the retina^5,6^. Within the retina, CDHR1 predominantly localizes to the junction between the inner and outer segments of both photoreceptors and is considered to have a crucial role in photoreceptor outer segment disc assembly^7,8^. Here, we report a case involving a Japanese family affected with mild (delayed onset) CRD in which a novel *CDHR1* variant was found using whole-exome sequencing (WES).

This research was approved by the Ethics Committee of Hamamatsu University School of Medicine. The protocol adhered to the tenets of the Declaration of Helsinki, and written informed consent was obtained from all patients.

The two affected siblings of the Japanese family were the proband (59 years of age; II-2 in Fig. 1a) and her elder brother (62 years of age; II-1 in Fig. 1a). An unaffected younger sister (55 years of age) is depicted as II-3 in Fig. 1a. Patients II-2 and II-1 were separately referred to our hospital at 41 and 54 years of age, respectively, with the chief complaint of night blindness, which had originally developed in their thirties. The best-corrected visual acuity (BCVA) in II-2 was 1.2 decimals in the right eye and 1.5 decimals in the left eye. The BCVA in II-1 was 1.0 decimals in both eyes. The proband confirmed that there was no family history of visual problems or symptoms in the other family members. Full-field electroretinograms (ERGs) revealed a generalized cone-rod pattern of dysfunction in both patients (Fig. 1b, c). More recently (at 58 years of age), the BCVA in II-2 had severely deteriorated to 0.03 decimals in either eye, while it had mildly deteriorated in II-1 to 0.6 decimals in the right eye and to 0.1 decimals in the left eye (at 62 years of age). At that time, fundus examination revealed macular atrophy in both patients (Fig. 1d, e) and additional diffuse retinal degeneration in II-2 (Fig. 1d). Fundus autofluorescence (FAF) showed hypo-autofluorescence in the macular area along with arcade vessels in both patients (Fig. 1f, g). It was more severe in II-2 (Fig. 1f). Optical coherence tomography (OCT) revealed apparent thinning of the retina and disruption of the ellipsoid zone in both patients (Fig. 1h, i), but retinal pigment epithelium was relatively preserved around the parafoveal area in II-1 (Fig. 1i). Goldmann visual fields exhibited central scotomas in both patients (Fig. 1j, k), and these scotomas were larger in II-2 (Fig. 1j). ERGs were nonrecordable under both scotopic and photopic conditions in II-2 (Fig. 1b), while reduced, but detectable, scotopic responses were obtained in II-1 (Fig. 1c). Their healthy sister (II-3) was asymptomatic with a normal BCVA, showed a healthy retinal structure with OCT, and had a normal retinal function with ERG (data not shown).

**Fig. 1:**
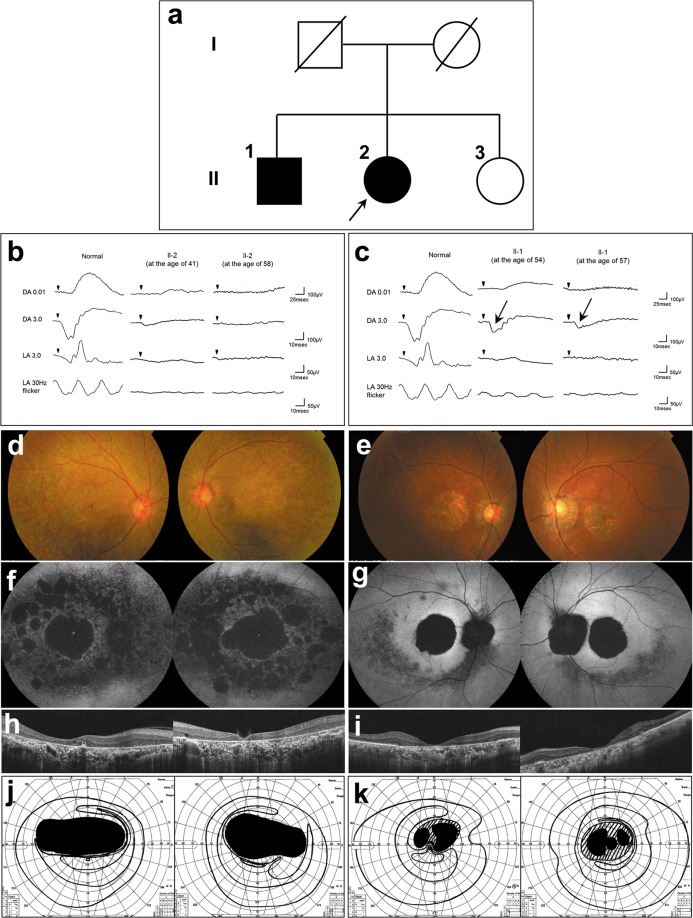
A pedigree of a Japanese family with CRD and the results of various ophthalmological examinations. **a** A pedigree of the Japanese family with CRD. The square boxes and circles denote male and female members, respectively; black symbols indicate affected individuals; and slashed symbols indicate deceased individuals. The proband is indicated with an arrow. **b**, **c** Electroretinograms of the proband (**b**) and her elder brother (**c**). DA, dark adaptation; LA, light adaptation. For each of the 2 patients, both the results at presentation and the more recent results are shown. The patient’s age at the time of examination is indicated. The scotopic responses in the elder brother’s ERG are indicated with arrows (**c**). A normal ERG pattern of a healthy individual (from our hospital) was added for comparison. **d**, **e** Fundus photographs of the proband (**d**) and her elder brother (**e**). **f**, **g** Fundus autofluorescence (FAF) of the proband (**f**) and her elder brother (**g**). **h**, **i** Optical coherence tomography (OCT) of the proband (**h**) and her elder brother (**i**). **j**, **k** Goldmann visual field of the proband (**j**) and her elder brother (**k**)

Peripheral blood leukocytes were collected from II-1, II-2, and II-3. The genomic DNA was extracted using the QIAamp DNA Blood Midi Kit (Qiagen, Hilden, Germany), and WES was performed on the DNA of II-1 and II-2 using the SureSelect XT Human All Exon V6 kit (Agilent Technologies, Santa Clara, CA, USA) and the NextSeq 500 system (Illumina, San Diego, CA, USA) as we previously reported^9^. The sequence reads were mapped to the human reference genome sequence (GRCh37/hg19) using Burrows-Wheeler Aligner software v 0.7.15. ANNOVAR software v 2016Feb01 was used to annotate the single nucleotide variants and the insertion-deletion polymorphisms. Among the rare nonsynonymous variants and the splice site variants, we screened variants in the 193 known inherited retinal disease genes listed in RetNet^TM^ (http://www.sph.uth.tmc.edu/RETNET/; accessed on 08 October 2017) because variants of these genes account for approximately 40% of the inherited retinal disease cases^10^. No other family members besides II-1 and II-2 were affected, suggesting autosomal recessive (ar) inheritance. Therefore, we performed an analysis based on the ar model. The steps that were used to filter the ar variants in this study are described in Supplementary Table S1.

Two heterozygous variants of *CDHR1* were evident in the two patients as follows: NM_033100.3(CDHR1_v001):c.1106dup [NP_149091.1:p.H370Afs*17] in exon 11 and NM_033100.3(CDHR1_v001):c.2027 T > A [NP_149091.1: p.I676N] in exon 16. The former was located between the 3rd and 4th cadherin domains, and the latter was located within the 6th cadherin domain (Supplementary Fig. S1c). The missense variant c.2027 T > A has been previously reported^11^ as a homozygous variant that causes ar retinitis pigmentosa (arRP). There has been no other report for c.2027 T > A causing CRD until now. The data implicate c.2027 T > A as the cause of CRD. The frameshift variant c.1106dup (p.H370Afs*17) was newly identified in this study in which a single nucleotide “A” at the 1106th position was duplicated. The variant is considered to result in nonsense-mediated mRNA decay.

Segregation analysis using Sanger sequencing further validated these variants. The analyses were performed using the KOD -plus- ver. 2 PCR Kit (Toyobo Co. LTD., Osaka, Japan), the BigDye Terminator v3.1 Cycle Sequencing Kit (Applied Biosystems, Foster City, CA, USA) and an ABI PRISM 3100 auto sequencer (Applied Biosystems). The c.2027 T > A (p.I676N) variant was found in II-1, II-2, and II-3. In contrast, the novel variant c.1106dup (p.H370Afs*17) was present in II-1 and II-2 but not in II-3 (Supplementary Fig. S1a, b). Therefore, we concluded that these two variants are pathogenic and caused CRD as a compound heterozygous state in both siblings.

Although the genotype was identical for the affected siblings, the phenotype severity differed. The more severe phenotype of the proband compared to II-1 was clearly evident by the clinical findings, which included fundus, OCT, visual field, and ERG (see above text and Fig. 1) examination. The age of presentation for night blindness at our hospital was younger for II-2 than that for II-1 (41 vs 54 years); this outcome also supports the more severe phenotype of the proband. After the onset of night blindness, the BCVA of both patients gradually deteriorated with age and a nearly identical rate of deterioration (see regression lines in Fig. 2).

**Fig. 2:**
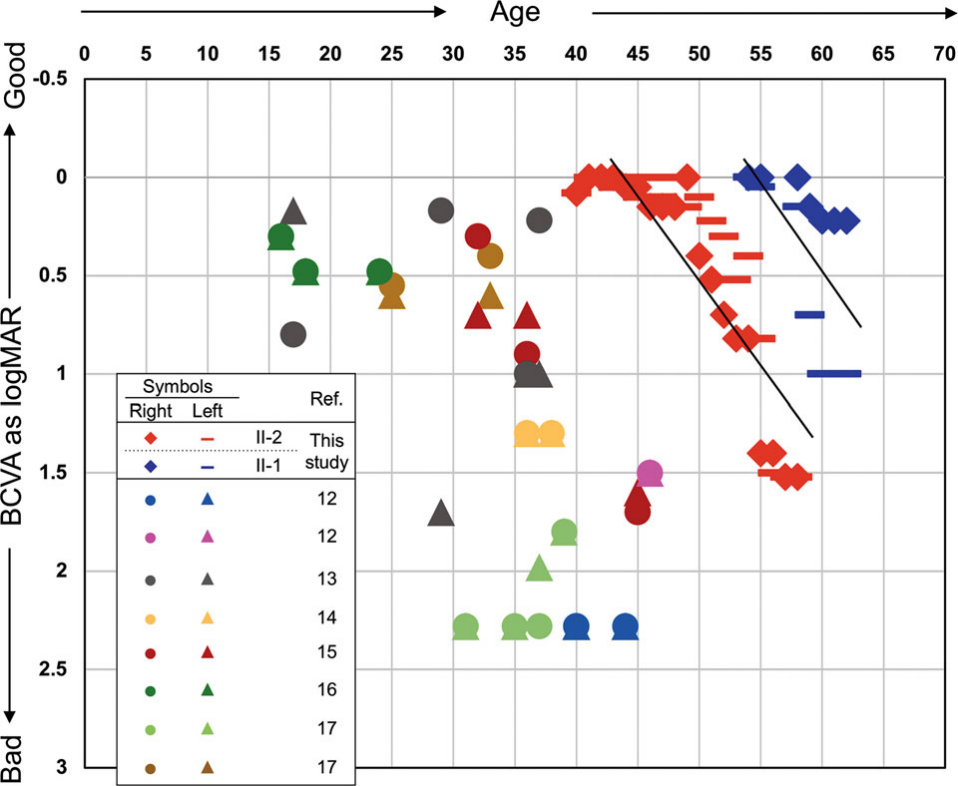
The best-corrected visual acuity (BCVA) of the patients in this study and the previously reported cases of CRD caused by *CDHR1* variants. The BCVAs (*y*-axis) of right and left eyes expressed as log values of the minimum angle of resolution (MAR) were plotted with the patient’s age (*x*-axis) at measurement. For the patients in this study, all of the multiple measurement results for all years were plotted, and linear regression lines were drawn. However, single data points were plotted for previously reported cases because only one value was recorded for most cases. For some exceptional patient cases in which 2 or more data points at different ages were described^12^, the data of the worst BCVA was used here. Cases without the BCVA data were excluded from this graph^18^. The symbols for each of the cases are indicated in the inset table. Multiple points with the same symbol indicate different patients from the same family

Although the siblings displayed different severities of CRD symptoms, their phenotypes were milder than those of typical CRD cases. Therefore, we performed a meta-analysis of the 24 previously reported CRD cases caused by *CDHR1* variants^12–18^ together with the siblings in this report (26 cases total). A plot of BCVA and age at presentation of the 26 cases (Fig. 2) confirmed that the majority of cases presented at younger ages than those of the siblings in this study. Next, we analyzed the possible correlation between variant type and each symptom. The variant type of “large-scale loss of protein” (LLP) was defined as including nonsense, frameshift, splicing, and large deletions. Twenty-two (84.6%) of the 26 cases involved the LLP/LLP combination, with the remaining four (15.4%) involving LLP/Missense (LLP/Mis). No case displayed the Missense/Missense (Mis/Mis) combination (Supplementary Table S2). Symptom information, including onset age, BCVA, ERG, color vision, visual field, OCT, fundus photographs, and FAF, were obtained for all cases. The severities of the symptoms were determined (Supplementary Table S3) as was the correlation between the severities and the variant combinations. The BCVA, color vision, fundus photographs, the visual field, and OCT tended to be milder in cases with LLP/Mis than those in cases with LLP/LLP (Supplementary Fig. S2(i)). However, the onset age, ERG, and FAF did not show any correlation (Supplementary Fig. S2(ii)). In particular, the findings of ERG (which is a reliable test to assess retinal cell function) did not correlate with the variant types. Additionally, the two siblings analyzed also displayed different severities in various symptoms despite having the same variant combination. These collective facts highlight the difficultly in understanding the relatively mild phenotype of the siblings according to the variant type combination of LLP/Mis.

In conclusion, we found a novel frameshift variant c.1106dup (p.H370Afs*17) in a Japanese family featuring two siblings with CRD. This variant is probably pathogenic, along with the known missense variant c.2027 T > A (p.I676N) previously reported in an arRP case. It is considered that the CRD in this family was caused by the combination of those heterozygous variants. A meta-analysis of the reported CRD cases caused by *CDHR1* variants indicated no clear-cut relationship between the symptom severity and the variant type.

## Supplementary information

Supplementary table S1: Filtering steps to select for autosomal recessive variants in this study

Supplementary table S2: The combination of variant type of <i>CDHR1</i> in CRD patients

Supplementary table S3: Symptom information of 26 cases of CRD and judgement of severity

Supplementary figure S1: Sequencing analysis for <i>CDHR1</i> gene in the family members with CRD and a protein domain structure of CDHR1 with the positions of identified variants

Supplementary figure S2(i): Correlation between each of clinical symptoms and variant type

Supplementary figure S2(ii): Correlation between each of clinical symptoms and variant type

## Data Availability

The relevant data from this data report are hosted at the Human Genome Variation Database at 10.6084/m9.figshare.hgv.2549 10.6084/m9.figshare.hgv.2552
