# Supplementary material for: A Japanese family with cone-rod dystrophy of delayed onset caused by a compound heterozygous combination of novel CDHR1 frameshift and known missense variants
Source: Hum Genome Var. 2019 Apr 12;6:18. doi: 10.1038/s41439-019-0048-8 (PMC6459921; doi:10.1038/s41439-019-0048-8)
Supplement: Supplementary file 1 — Supplementary table S1: Filtering steps to select for autosomal recessive variants in this study [file 41439_2019_48_MOESM1_ESM.pdf]

Supplementary Table S1: The steps used to filter for autosomal recessive variants in this study.

**Filtering criteria**	**Proband/ II-2**	**Elder brother/ II-1**
Homozygous or heterozygous variants in affected individuals[a]	24162	24251
193 autosomal recessive genes listed in the RetNet database[b]	388	370
Allele frequency of ≤ 0.005 in the public SNP databases[c]	7	4
Two or more variants in one gene	2 (*CDHR1*)	2 (*CDHR1*)

[a]The variants localized in the coding exonic regions and in the intronic regions within 5 bp of the exon-flanking boundaries (± 5 bp) were included.

[b]The reported 193 genes responsible for autosomal recessive inherited retinal dystrophy (accessed 8th October 2017 (https://sph.uth.edu/retnet/)).

[c]The following databases were used: 1000 Genomes database (http://www.1000genomes.org/), Exome Aggregation Consortium database (http://exac.broadinstitute.org/), Human Genetic Variation Database (HGVD) (http://www.genome.med.kyoto-u.ac.jp/SnpDB/), and Tohoku Medical Megabank Organization database (ToMMo) (https://ijgvd.megabank.tohoku.ac.jp/).
