# Supplementary material for: A Japanese family with cone-rod dystrophy of delayed onset caused by a compound heterozygous combination of novel CDHR1 frameshift and known missense variants
Source: Hum Genome Var. 2019 Apr 12;6:18. doi: 10.1038/s41439-019-0048-8 (PMC6459921; doi:10.1038/s41439-019-0048-8)
Supplement: Supplementary file 2 — Supplementary table S2: The combination of variant type of CDHR1 in CRD patients [file 41439_2019_48_MOESM2_ESM.pdf]

Supplementary Table S2: The combination of variant types of *CDHR1* in CRD patients.

Genotype-disease analysis in CRD		
	**Patient numbers**	**Percentage**
**LLP/LLP**	22	84.6%
**LLP/Mis**	4	15.4%
**Mis/Mis**	0	0%

The variant types of *CDHR1* in CRD patients described in the literature[12-18] were analyzed for type (LLP or Missense). The siblings analyzed in this study were also included. See the text for the definition of LLP. LLP/LLP means homozygous or compound heterozygous of LLP, and LLP/Mis means compound heterozygous of LLP and a missense variant.

CRD: Cone-rod dystrophy
LLP: Large-scale loss of protein
Mis: Missense
