# Supplementary material for: A Japanese family with cone-rod dystrophy of delayed onset caused by a compound heterozygous combination of novel CDHR1 frameshift and known missense variants
Source: Hum Genome Var. 2019 Apr 12;6:18. doi: 10.1038/s41439-019-0048-8 (PMC6459921; doi:10.1038/s41439-019-0048-8)
Supplement: Supplementary file 3 — Supplementary table S3: Symptom information of 26 cases of CRD and judgement of severity [file 41439_2019_48_MOESM3_ESM.pdf]

Supplementary Table S3: The symptom information of 26 cases of CRD and the judgement of severity.

Patient number	Patient ID	Sex	Clinical diagnosis	Genotype		Onset age	Age at Examination	BCVA (as logMAR)	Color vision	ERG	Visual field	Fundus	FFA	OCT	Reference
				Nucleotide change	Protein change										
1	II-2/Proband/ Japanese family	F	CRD	Heterozyous c.1106dup c.2027T>A	p.H370Afs*17 p.I676N	30	41	OD: -0.10 OS: -0.20	Can pass Ishihara test	Generalized cone-rod pattern of dysfuntion	–	–	–	–	This study
								OU: Mild	Mild	Moderate	_	_	_	_	
						Mild	59	OU: 1.52	Severely impaied	Non-recordable under both scotopic and photopic conditions	Larger central scotoma	Macular atrophy with additional diffuse retinal degeneration	Hypo-autofluorescence in the macular area along with arcade vessels. More area involved.	Apparent thinning of the retina and disruption of the ellipsoid zone.	
								OU: Severe	Severe	Severe	Severe	Severe	Severe	Moderate	
2	II-1/Elder brother/ Japanese family	M	CRD	Heterozyous c.1106dup c.2027T>A	p.H370Afs*17 p.I676N	30	54	OU: 0.00	Some color vision preserved	Generalized cone-rod pattern of disfuntion	–	–	–	–	This study
								OU: Mild	Moderate	Moderate	_	_	_	_	
						Mild	62	OD: 0.2 OS:1.00	Severely impaied	ERGs reduced but detectable scotopic responses	Central scotoma	Macular atrophy	Hypo-autofluorescence in the macular area along with arcade vessels	Apparent thinning of the retina and disruption of the ellipsoid zone; RPE was relatively preserved around the parafoveal area.	
								OD: Mild OS: Severe	Severe	Moderate	Moderate	Moderate	Moderate	Mild	
3	IV-2/Family 1	F	Retinal degeneration	Homozygous c.338delG	p.G113AfsX1	Late teenage	44	OU: 2.28	Severe color vision defect	ERG showed both cone and rod responses.	Not available	Vessel attenuation, diffuse RPE changes, and dense bone spicule pigment migration in the retinal periphery and atrophy both at the macula and in the periphery	Not available	Not available	[12]
						Moderate		OU: Severe	Severe	Moderate	_	Severe	_	_	
4	IV-4/Family 1	M	Retinal degeneration	Homozygous c.338delG	p.G113AfsX1	Late teenage	40	OU: 2.28	Severe color vision defect	ERG showed both cone and rod responses.	Not available	Vessel attenuation, diffuse RPE changes, and dense bone spicule pigment migration in the retinal periphery and atrophy both at the macula and in the periphery	Not available	Not available	[12]
						Moderate		OU: Severe	Severe	Moderate	_	Severe	_	_	
5	IV-5/Family 1	F	Retinal degeneration	Homozygous c.338delG	p.G113AfsX1	Late teenage	40	OU: 2.28	Severe color vision defect	ERG showed both cone and rod responses.	Not available	Vessel attenuation, diffuse RPE changes, and dense bone spicule pigment migration in the retinal periphery and atrophy both at the macula and in the periphery	Not available	Not available	[12]
						Moderate		OU: Severe	Severe	Moderate	_	Severe	_	_	
6	IV-6/Family 1	M	Retinal degeneration	Homozygous c.338delG	p.G113AfsX1	Late teenage	40	OU: 2.28	Severe color vision defect	Cone–rod pattern of disease	Not available	Vessel attenuation, diffuse RPE changes, and dense bone spicule pigment migration in the retinal periphery and atrophy both at the macula and in the periphery	Not available	Not available	[12]
						Moderate		OU: Severe	Severe	Moderate	_	Severe	_	_	
7	II-1/Family 2	M	Retinal degeneration	Homozygous c.1463delG	p.G488AfsX20	18	46	OU: 2.28	No color vision	Detectable rod or cone responses in either eye	V4e isopter in green and III4e in red: there are peripheral islands of residual vision and no response centrally to the largest and brightest target (V4e) in the right eye and small areas of response in the left eye.	Circular patches of pigment epithelial atrophy both at the macula and in the periphery associated with pigment migration and vessel attenuation	Not available	Not available	[12]
						Moderate		OU: Severe	Severe	Moderate	Severe	Severe	_	_	
8	II-4/Faroe Islands CRD fam.	M	CRD	Homozygous c.524dupA	p.Q175QfsX47	17	29	OD: 0.17 OS: 1.7	Not available	Undetectable	Only narrow peripheral remnants left in the lower visual fields and a paracentral preserved island of 10°	Diffuse atrophy of RPE; extensive sheen in the macular region; numerous, polymorphic, intraretinal hyperpigmentations in the upper temporal midperiphery.	Not available	Not available	[13]
						Moderate		OD: Mild OS: Severe	_	Severe	Severe	Moderate	_	_	
9	II-6/Faroe Islands CRD fam.	F	CRD	Homozygous c.524dupA	p.Q175QfsX47	17	36	OU: 1.00	Severely impaired	Markedly delayed and reduced rod and cone responses	Normal or slightly constricted outer field limits. A central scotoma measuring 5° was found.	Irregular foveal degeneration and normal arteriolar caliber	Not available	Not available	[13]
						Moderate		OU: Severe	Severe	Moderate	Moderate	Moderate	_	_	
10	II-10/Faroe Islands CRD fam.	M	CRD	Homozygous c.524dupA	p.Q175QfsX47	17	37	OD: 0.22 OS: 1.00	Severely impaired	Markedly delayed and reduced rod and cone responses	Normal or slightly constricted outer field limits.	Irregular RPE atrophy in the macular region, attenuated arterioles and paleness of the optic nerve head	Not available	Not available	[13]
						Moderate		OD: Mild OS: Severe	Severe	Moderate	Moderate	Moderate	_	_	
11	IV-2/Faroe Islands CRD fam.	M	CRD	Homozygous c.524dupA	p.Q175QfsX47	15	17	OD: 0.8 OS: 0.17	Not available	Undetectable	Not available	Foveal atrophy and attenuated arterioles	Not available	Not available	[13]
						Moderate		OD: Severe OS: Mild	_	Severe	_	Moderate	_	_	
12	IV-2/TB-127	F	CRD	Homozygous c.1485+2T>G	Elimination of intron 13 donor splice site	20	36	OU: 1.3	No color vision	Cone responses absent, and reduced rod responses	Bilateral deep large central scotomas with significant general reduction of sensitivity in both eyes	Pigmentary deposits localized mainly in the peripheral retina, a thinned and atrophic macular region, retinal vessel attenuation.	Not available	Severe thinning of the macula, with irregular RPE	[14]
						Moderate		OU: Severe	Severe	Moderate	Severe	Moderate	_	Severe	
13	IV-3/TB-127	F	CRD	Homozygous c.1485+2T>G	Elimination of intron 13 donor splice site	20	38	OU: 1.3	No color vision	Cone responses absent, and reduced rod responses	Bilateral deep large central scotomas with significant general reduction of sensitivity in both eyes	Pigmentary deposits localized mainly in the peripheral retina, a thinned and atrophic macular region, retinal vessel attenuation.	Not available	Macular thinning and loss of foveal contour	[14]
						Moderate		OU: Severe	Severe	Moderate	Severe	Moderate	_	Moderate	
14	IV-1/ Palestinian family	F	Retinal degeneration	Homozygous c.1381C>T	p.Q461X	Childhood	45	OD: 1.7 OS: 1.6	Some color vision preserved	Rod and cone responses were severely reduced	Peripheral islands of vision temporally and inferiorly with central vision loss	Bull's eye pattern of RPE atrophy in the macula and circular patches of RPE atrophy anterior to the arcades, with retinal vascular attenuation and bone spicule pigmentation.	Not available	Thinning and loss of the outer nuclear, IS, and OS layers.	[15]
						Severe		OU: Severe	Moderate	Severe	Severe	Severe	_	Severe	
15	IV-3/ Palestinian family	F	Retinal degeneration	Homozygous c.1381C>T	p.Q461X	Childhood	36	OD: 0.9 OS: 0.7	No color vision	Rod and cone responses were severely reduced	Severe field constriction with small central island	Bull's eye pattern of RPE atrophy in the macula and circular patches of RPE atrophy anterior to the arcades, with retinal vascular attenuation and bone spicule pigmentation.	Not available	Thinning and loss of the outer nuclear, IS, and OS layers. The outer retinal layers were relatively preserved around the anatomic fovea.	[15]
						Severe		OU: Moderate	Severe	Severe	Severe	Moderate	_	Moderate	
16	IV-4/ Palestinian family	F	Retinal degeneration	Homozygous c.1381C>T	p.Q461X	Childhood	32	OD: 0.3 OS: 0.7	Some color vision preserved	Rod and cone responses were severely reduced	Severe field constriction with small central island	Bull's eye pattern of RPE atrophy in the macula and circular patches of RPE atrophy anterior to the arcades, with retinal vascular attenuation and bone spicule pigmentation.	Not available	Thinning and loss of the outer nuclear, IS, and OS layers. The outer retinal layers were relatively preserved around the anatomic fovea.	[15]
						Severe		OD: Mild OS: Moderate	Moderate	Severe	Severe	Moderate	_	Moderate	
17	II-2/GC18832	F	CRD	Homozygous c.1463delG	p.G488fs	18	24	OU: 0.48	Some color vision preserved	Undetectable PERG; Scotopic bright flash ERG is markedly delayed and subnormal; Severely delayed and reduced cone responses.	OU: Relatively large central scotoma; OS: smaller paracentral islands of sensitivity loss.	RPE changes at the macula; normally appearing peripheral retina	Speckled hypo-autofluorescence in the macular region.	Disruption of the photoreceptor and RPE layers at the fovea	[16]
						Moderate		OU: Mild	Moderate	Severe	Severe	Mild	Mild	Mild	
18	II-3/GC18832	M	CRD	Homozygous c.1463delG	p.G488fs	15	18	OU: 0.48	Severely impaied	Undetectable PERG; Scotopic bright flash ERG is markedly delayed and subnormal; Severely delayed and reduced cone responses.	OU: Normal Fields	Subtle RPE atrophic changes at the center of both maculae	Normal fundus autofluorescence	Not available	[16]
						Moderate		OU: Mild	Severe	Severe	Mild	Mild	Mild	_	
19	II-6/GC18832	F	CRD	Homozygous c.1463delG	p.G488fs	15 1/2	16	OU: 0.3	Only demo Ishihara plate in both eyes	PERG: severely reduced; Scotopic bright flash ERG is moderately subnormal; Severely delayed & reduced cone responses.	OD: a left central and paracentral relative scotomata to static stimuli. OS: A small relative scotoma.	Elevated, pigmented macular lesion; normally appearing peripheral retina	A ring of hyper-autofluorescence surrounding a central bull's eye lesion.	A localized dome-shaped foveal elevation in both eyes involving the RPE layer, which also had an uneven reflectivity	[16]
						Moderate		OU: Mild	Severe	Severe	Moderate	Mild	Mild	Moderate	
20	1/Family A	M	CRD	Homozygous c.2522_2528 delTCTCTGA	p.I841Sfs*119	9	39	OU: 1.81	Severely impaied	Rod & cone responses nearly extinguished	Larger central visual field defect	Macular atrophy, pigment loss, tigroid appearance, optic nerve disc pallor, retinal vessel attenuation.	Hypo-autofluorescence in the macular area along with arcade vessels. More area involved.	Disturbance in cone outer segments and RPE layer was intense.	[17]
						Severe		OU: Severe	Severe	Severe	Severe	Severe	Severe	Severe	
21	2/Family A	M	CRD	Homozygous c.2522_2528 delTCTCTGA	p.I841Sfs*119	14	37	OD: 2.28 OS: 1.98	Severely impaied	Rod & cone responses extinguished	Not measurable	Macular atrophy, pigment loss, optic nerve disc pallor, retinal vessel attenuation.	Hypo-autofluorescence in the macular area along with arcade vessels. More area involved.	Disturbance in cone outer segments and RPE layer was intense.	[17]
						Moderate		OU: Severe	Severe	Severe	Severe	Severe	Severe	Severe	
22	3/Family A	M	CRD	Homozygous c.2522_2528 delTCTCTGA	p.I841Sfs*119	12	31	OU: 2.28	Severely impaied	Rod & cone responses extinguished	Not measurable	Macular atrophy, pigment loss, optic nerve disc pallor, retinal vessel attenuation.	Not available	Disturbance in cone outer segments and RPE layer was intense.	[17]
						Moderate		OU: Severe	Severe	Severe	Severe	Severe	_	Severe	
23	4/Family A	F	CRD	Homozygous c.2522_2528 delTCTCTGA	p.I841Sfs*119	14	35	OU: 2.28	Severely impaied	Rod & cone responses extinguished	Not measurable	Macular atrophy, pigment loss, tigroid appearance, optic nerve disc pallor, retinal vessel attenuation.	Hypo-autofluorescence in the macular area along with arcade vessels. More area involved.	Disturbance in cone outer segments and RPE layer was intense.	[17]
						Moderate		OU: Severe	Severe	Severe	Severe	Severe	Severe	Severe	
24	1/Family B	M	CRD	Heterozyous c.1448A>G c.2522_2528 delTCTCTGA	p.E483G p.I841Sfs*119	Childhood	33	OD: 0.4 OS: 0.6	Color vision defect present	Rod & cone responses extinguished	Central scotoma	Geographic atrophy of the macula, pigment loss, tigroid appearance, retinal vessel attenuation.	Not available	Mild disturbance in cone outer segments and RPE layer.	[17]
						Severe		OD: Mild OS: Moderate	Mild	Severe	Mild	Mild	_	Mild	
25	2/Family B	F	CRD	Heterozyous c.1448A>G c.2522_2528 delTCTCTGA	p.E483G p.I841Sfs*119	Teenage	25	OD: 0.54 OS: 0.6	Color vision defect present	Rod and cone ERG not detectable; Mixed cone-rod ERG reduced.	Relatively smaller central scotoma	Changes of the retinal pigment epithelium in the macula, optic nerve disc pallor, no pigmentary changes in the periphery, retinal vessel attenuation.	Not available	Mild disturbance in cone outer segments and RPE layer.	[17]
						Moderate		OU: Moderate	Mild	Severe	Mild	Mild	_	Mild	
26	II-4/M341	F	Retinal dystrophy/ MD/CRD	Homozygous c.T1641A	p.Y547*	24	45	Markly reduced	Severely impaied	Markedly reduced rod and cone responses	Not available	Attenuated vessels, absence of the foveal reflex, punctated salt- and pepper-like appearance, circular patches of RPE atrophy both at the macula and in the periphery with associated peripheral pigment migration	A hyper-autofluorescent ring surrounding a central area of hypo-autofluorescence and an atrophic macular region	Not available	[18]
						Moderate		Severe	Severe	Severe	_	Severe	Severe	_	

The information of 8 symptoms (onset age, BCVA, ERG, color vision, visual field, OCT, fundus photograph, and FAF) for each patient described in the literature[12-18] and the siblings analyzed in this study were extracted. The judgment of severity was performed as follows:

Onset age: 21 – 30 years/or greater (mild), 11 – 20 years (moderate), 0 – 10 years (severe);

BCVA (as logMAR): -0.2 – 0.48 (mild), 0.49 – 0.9 (moderate), 0.91 – or greater (severe);

Color vision defect: Can pass more than 10 Ishihara plates (mild), can pass less than 10 Ishihara plates (moderate), cannot read a demo plate (severe);

ERG: Both cone and rod ERG present and slightly reduced (mild); Either cone or rod ERG undetectable and the other one reduced but detectable (moderate); Both cone and rod responses undetectable/severely reduced (severe);

Fundus: Changes of RPE at the macula, vessel attenuation and less pigmentary change in the periphery (mild), early macular atrophy and sparse bone spicule pigment (moderate), obvious macular atrophy and dense bone spicule pigment (severe);

Visual field: Normal field or small paracentral scotoma/slightly constricted outer field (mild); medium central scotoma/constricted outer field (moderate), large central scotoma/severe field constriction (severe);

FAF: Normal fundus autofluorescence and slight hypo-autofluorescence in the macular area (mild), hypo-autofluorescence in the macular area along with arcade vessels (moderate), larger area with hypo-autofluorescence in the macular area and arcade vessels (severe);

OCT: The RPE was relatively preserved around the parafoveal area (mild), the apparent thinning of the retina and disruption of the ellipsoid zone (moderate), the macular thinning and loss of foveal contour (severe).

Abbreviations: CRD, cone-rod dystrophy; BCVA, best-corrected visual acuity; OD, oculus dexter (right eye); OS, oculus sinister (left eye); OU, oculus uterque (both eyes); ERG, electroretinogram; PERG, pattern electroretinogram; RPE, retinal pigment epithelium; FAF, fundus autofluorescence; OCT, optical coherence tomography; IS, inner segment; OS, outer segment.
