# Supplementary material for: A Japanese family with cone-rod dystrophy of delayed onset caused by a compound heterozygous combination of novel CDHR1 frameshift and known missense variants
Source: Hum Genome Var. 2019 Apr 12;6:18. doi: 10.1038/s41439-019-0048-8 (PMC6459921; doi:10.1038/s41439-019-0048-8)
Supplement: Supplementary file 4 — Supplementary figure S1: Sequencing analysis for CDHR1 gene in the family members with CRD and a protein domain structure of CDHR1 with the positions of identified variants [file 41439_2019_48_MOESM4_ESM.pdf]

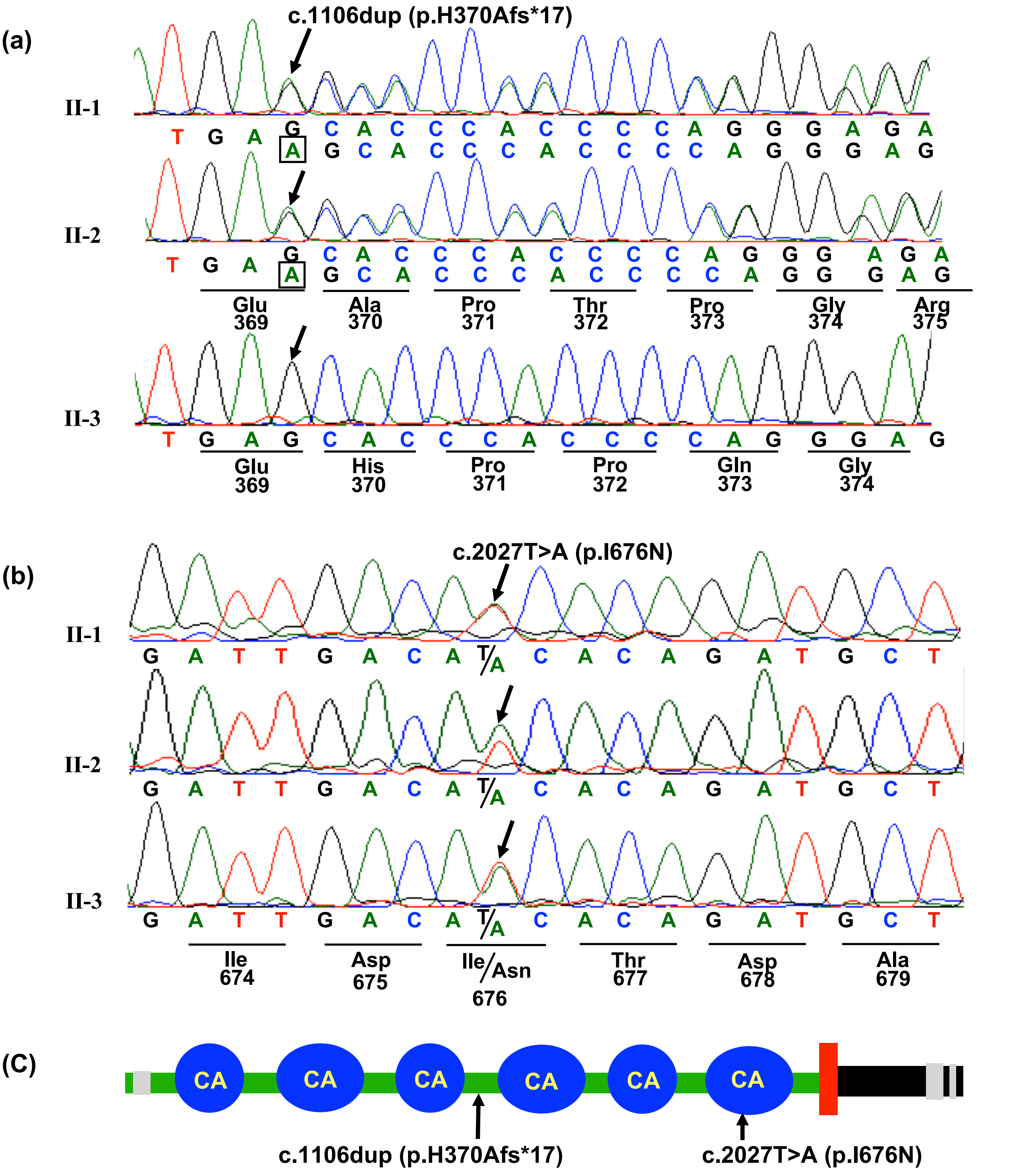

Supplementary Figure S1: The sequencing analysis for the *CDHR1* gene in the family members with CRD and the protein domain structure of CDHR1 with the positions of the identified variants.

(a) Electropherograms of all siblings with Sanger DNA sequencing of exon 11 of *CDHR1* encompassing the c.1106dup region showing a single nucleotide 'A' duplication and a frameshift in the two affected siblings. The sequence wave pattern in the region with the frameshift variant was identified as a mixed pattern of variant and normal waves. The predicted amino acid sequences and numbers are shown along with nucleotide sequences. Histidine at amino acid position 370 is the first amino acid that was changed to alanine in the variant allele, and the premature stop codon TGA was created at 17 codons downstream from that position. The frameshift variant (c.1106dup) was not identified in the unaffected younger sister. (b) Electropherograms of all siblings with Sanger DNA sequencing for exon 16 of the *CDHR1* encompassing the c.2027T>A region. All sibling members showed the same heterozygous change from isoleucine to asparagine at codon 676 (p.I676N). (c) A protein domain structure of CDHR1 with the positions of the identified variants. The extracellular cadherin repeats (CA), transmembrane domain, intracellular domain and low complexity region are indicated as blue, red, black and ash, respectively.
