# Supplementary material for: A Japanese family with cone-rod dystrophy of delayed onset caused by a compound heterozygous combination of novel CDHR1 frameshift and known missense variants
Source: Hum Genome Var. 2019 Apr 12;6:18. doi: 10.1038/s41439-019-0048-8 (PMC6459921; doi:10.1038/s41439-019-0048-8)
Supplement: Supplementary file 5 — Supplementary figure S2(i): Correlation between each of clinical symptoms and variant type [file 41439_2019_48_MOESM5_ESM.pdf]

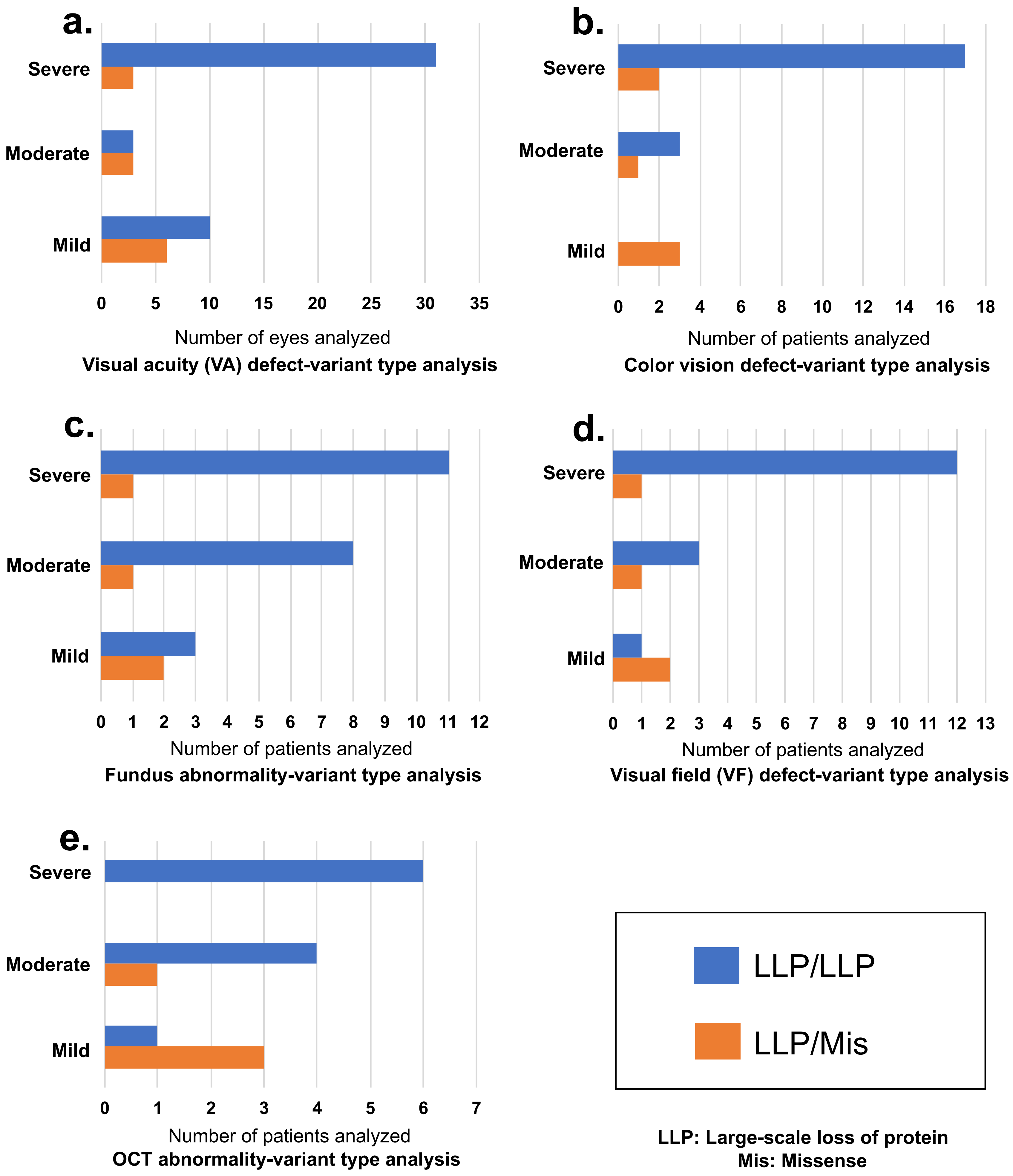

Supplementary figure S2(i): The correlation between each of the clinical symptoms and the variant types.

The judged severity of each of the clinical symptoms of the CRD patients extracted from the literature (Supplementary Table S3) was analyzed for the correlation with the combination of variant type (LLP/LLP or LLP/Mis).

(i) In BCVA (a), the color vision (b), fundus photograph (c), visual field (d), and OCT (e) results showed a more relative number of patients with the LLP/LLP combination than that with the LLP/Mis.
